# A neonatal rat model of pulmonary vein stenosis

**DOI:** 10.1186/s13578-023-01058-8

**Published:** 2023-06-19

**Authors:** Debao Li, Lisheng Qiu, Haifa Hong, Hao Chen, Peibin Zhao, Yingying Xiao, Hao Zhang, Qi Sun, Lincai Ye

**Affiliations:** 1 Department of Thoracic and Cardiovascular Surgery, School of Medicine, Shanghai Children’s Medical Center, Shanghai Jiao Tong University, 1678 Dongfang Road, Shanghai, 200127 China; 2 Institute of Cardiovascular Development and Translational Medicine, Children’s Heart Center, The Second Affiliated Hospital and Yuying Children’s Hospital, Wenzhou Medical University, Wenzhou, 325027 China; 3 Department of Thoracic and Cardiovascular Surgery, School of Medicine, Shanghai Children’s Hospital, Shanghai Jiao Tong University, Shanghai, China; 4 Shanghai Institute for Pediatric Congenital Heart Disease, Shanghai Children’s Medical Center, Shanghai Jiao Tong University School of Medicine, 1678 Dongfang Road, Shanghai, 200127 China; 5 Institute of Pediatric Translational Medicine, School of Medicine, Shanghai Children’s Medical Center, Shanghai Jiao Tong University, Shanghai, China

**Keywords:** Pulmonary vein stenosis, Intimal hyperplasia, Pulmonary arterial hypertension, Right ventricular hypertrophy, Congenital heart disease, Lung remodeling

## Abstract

**Objectives:**

Pulmonary vein stenosis (PVS), one of the most challenging clinical problems in congenital heart disease, leads to secondary pulmonary arterial hypertension (PAH) and right ventricular (RV) hypertrophy. Due to the lack of a rodent model, the mechanisms underlying PVS and its associated secondary effects are largely unknown, and treatments are minimally successful. This study developed a neonatal rat PVS model with the aim of increasing our understanding of the mechanisms and developing possible treatments for PVS.

**Methods:**

PVS was created at postnatal day 1 (P1) by banding pulmonary veins that receive blood from the right anterior and mid lobes. The condition was confirmed using echocardiography, computed tomography (CT), gross anatomic examination, hematoxylin and eosin (H&E) staining, fibrosis staining, and immunofluorescence. Lung and RV remodeling under the condition of PVS were evaluated using H&E staining, fibrosis staining, and immunofluorescence.

**Results:**

At P21, echocardiography revealed a change in wave form and a decrease in pulmonary artery acceleration time—indicators of PAH—at the transpulmonary valve site in the PVS group. CT at P21 showed a decrease in pulmonary vein diameter in the PVS group. At P30 in the PVS group, gross anatomic examination showed pulmonary congestion, H&E staining showed wall thickening and lumen narrowing in the upstream pulmonary veins, and immunofluorescence showed an increase in the smooth muscle layers in the upstream pulmonary veins. In addition, at P30 in the PVS group, lung remodeling was evidenced by hyperemia, thickening of pulmonary small vessel walls and smooth muscle layers, and reduction of the number of alveoli. RV remodeling was evidenced by an increase in RV free wall thickness.

**Conclusions:**

A neonatal rat model of PVS was successfully established, showing secondary lung and RV remodeling. This model may serve as a useful platform for understanding the mechanisms and treatments for PVS.

**Supplementary Information:**

The online version contains supplementary material available at 10.1186/s13578-023-01058-8.

## Introduction

Although pulmonary vein stenosis (PVS) in the pediatric population is a rare disease, affected individuals all die, with the literature reporting mortality rates as high as 60% by 2 years after diagnosis [1, 2]. PVS has two important characteristics that make treating it extremely challenging: (1) recurrence despite anatomic intervention at the site of the stenosis and (2) progression of the stenosis to previously unaffected veins [3, 4]. As a result, PVS leads to secondary and relentless pulmonary arterial hypertension (PAH) and right ventricular (RV) hypertrophy, which cause children to die [5, 6].

Apart from surgical intervention, current medications focus on the inhibition of pulmonary vein intimal hyperplasia, and are based on histopathological changes unique to PVS that have been identified in PVS samples from humans or piglet models [7–10]. However, the results of such medications are disappointing, with 0% survival rate and mild to moderate side effects [7–10]. Moreover, compared with rodent models, piglet models are expensive, time-consuming, and difficult to manipulate genetically. In addition, a team including a surgeon, physician, and anesthesiologist is required to construct a piglet model of PVS [8, 10]. Due to these limitations, the underlying mechanisms that account for the recurrence and progression of stenosis are poorly understood. Furthermore, knowledge is limited about the possible unique mechanisms underlying the secondary effects of PVS, as well as about whether there is a feedback loop between the condition and its secondary effects [11, 12]. To help address these problems and to provide new insights into the treatment of PVS, we introduced a neonatal rat model of PVS.

## Materials and methods

### Study design

**Fig. 1:**
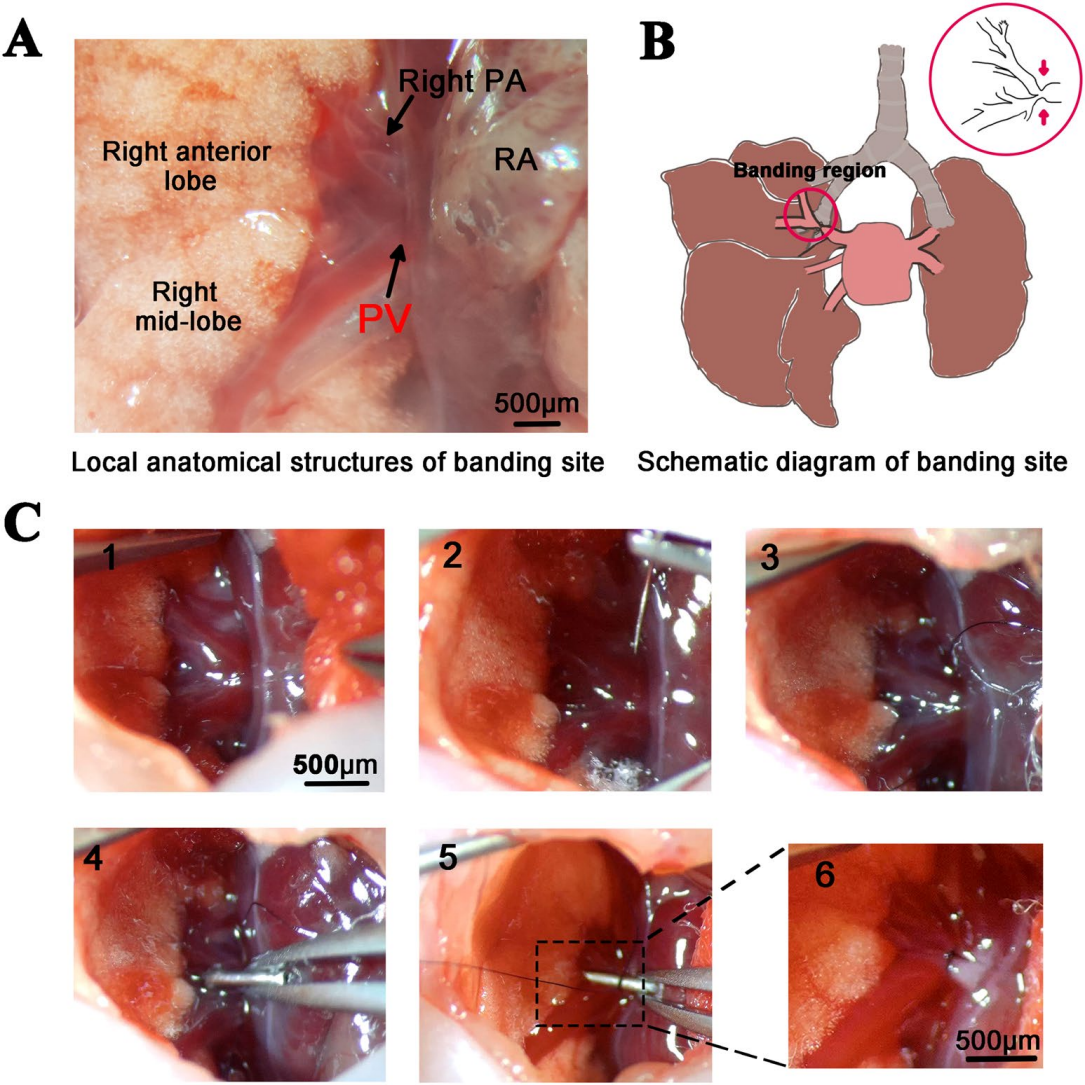
Surgical process of PV banding. (A) Local anatomical structures of banding site. (B) Schematic diagram of banding site. (C) Key steps of PV banding. 1: Expose right anterior and middle PV; 2, 3: Place a nylon thread; 4: Place a needle; 5: Tie the thread and needle and then remove the needle; 6: Create constricted PV lumen. *PV*: pulmonary vein; *RA*: right atrium; *PA*: pulmonary artery

This study employed 80 rat pups, with half receiving the pulmonary vein (PV) banding surgery, and the others undergoing sham surgery. The local anatomic structures of the PV and the banding site are shown in Fig. 1A and B. The surgery was performed on neonatal rat pups at postnatal day 1(P1) and the surgical procedures are shown in Fig. 1C, as well as Additional file 3: Video S1. To validate PVS and its secondary effects, we used echocardiography, computed tomography (CT), gross anatomic examination, hematoxylin and eosin (H&E) staining, and immunofluorescence at P21 and P30.

### Animals

The protocols in this investigation were in compliance with the guidelines for the Care and Use of Laboratory Animals and approved by the Animal Care and Use Committee of Shanghai Children’s Medical Center. PVS and sham operations were performed on neonatal Sprague–Dawley rats on P1. Pregnant rats were purchased from Jihui Laboratory Animal Breeding Co., Ltd. (Shanghai, China). At P1, the neonates (both males and females) were randomized to two groups of 40 rats each: the PVS and sham groups. Six rats from each group, or a total of 12, were randomly selected for echocardiography examination and CT evaluation at P21. At P30, all of the rats were sacrificed for gross anatomic examination, H&E staining, and immunofluorescence.

### Surgical protocol

PVS surgery was performed on the neonatal rats at P1 (see Additional file 3: Video S1). As shown in Fig. 1A and B, the right pulmonary anterior and middle veins are adjacent and anastomosing before entering the left atrium. Therefore, we banded the right pulmonary anterior and middle veins at the anastomosing site to create a stenosis. Neonates were anesthetized using approximately 3 min of direct ice-cooling, which led to asystole and reversible apnea and prevented excessive blood loss during surgery [13, 14]. The rats were then transferred to an ice bed and fixed in the supine position. As shown in the graphical abstract and Fig. 1C, following a transverse skin incision, a horizontal thoracotomy at the third intercostal space was performed by dissecting the intercostal muscles and cutting the sternum under a stereomicroscope. After carefully exposing the right anterior and middle PV (Fig. 1C(1)), a 12–0 nylon thread was positioned at the bottom of the PV, and a 30-gauge needle (0.35 mm in diameter) was placed next to the PV (Fig. 1C(2–4)). The PV and needle were then tied together with the thread (Fig. 1C(5)). After the needle was removed, a fixed constricted opening equal to the diameter of the needle remained (Fig. 1C(6)), determining the degree of stenosis. The sternum and thoracic wall were then sutured with a 9–0 nylon thread layer by layer. Afterward, the neonates were removed from the ice bed and placed under a 37 °C heat plate to warm for several minutes until natural movements and a red/pink complexion were achieved. The neonates were then returned to their mothers. The entire procedure lasted less than 10 min. The sham group underwent the same procedure except for the banding step.

### Transthoracic echocardiography

At P21, rats were anesthetized with isoflurane (isoflurane/oxygen: 5% induction, 1.5%-2.0% maintenance) and placed in a supine position. Echocardiograms were analyzed with a Vevo 2100 imaging system (Visual Sonics, Toronto, Ontario, Canada) equipped with a 25-MHz transducer (MS400 MicroScan transducer; Visual Sonics). In addition, a long-axis view of the PA was used to measure the PA acceleration time by pulsed-wave Doppler. All measurements were performed blinded by a single experienced ultrasound technician and were recorded as the average of three consecutive cardiac cycles.

### CT

At P21, after the rats were anesthetized following the same method above, a 2-ml dose of Iodixanol (GE Healthcare, USA) was injected from the tail vein, the animals were euthanized and then scanned with the Skyscan 1176 micro-CT imaging system (Bruker, Kontich, Belgium) using a spatial resolution of 35 μm (1-mm aluminum filter, 50 kV, 200 μA). The images were processed using CTVox (Version 3.3.0r1403, Bruker MicroCT, Kontich, Belgium) and DataViewer (Version 1.5.4.6, Bruker MicroCT, Kontich, Belgium) software. The coronal section was selected to compare the diameter of the right PV in both groups.

### Histology

At P30, the rats were sacrificed for gross anatomic examination, H&E staining, Masson trichrome staining, EVG staining, and immunofluorescence. The main branches of right PVs (upstream PVs) were dissected carefully under a stereomicroscope. The lungs were fixed and inflated by instilling the tissue fixation fluid through the trachea (0.2 mL/10 g, 5 cm H2O). Then, the lungs and hearts were removed and rinsed in phosphate-buffered saline solution. All of the tissues (right PVs, lungs, and hearts) were fixed in 10% paraformaldehyde overnight at room temperature, then dehydrated in an ethanol series, embedded in paraffin, and sectioned into 6-µm slices.

H&E staining was performed with an H&E kit (C0105M, Beyotime Biotech, Shanghai, China) according to the manufacturer’s instructions. The number of alveoli was evaluated by calculating mean linear intercepts (MLI) using the ImageJ software (www.rsb.info.nih.gov/ij; U.S. National Institutes of Health, Bethesda, MD, USA). To calculate the MLI, grids of horizontal and vertical lines were placed in the 20 × field of the lung section. The total number of times the lines intersected with the alveoli and the total length of the grids were recorded, then used to calculate MLI in μm, as follows: MLI = (total grid length/total number of intersections) [15, 16]. In addition, the thickness of RV free wall, smooth muscle layer of PV, and smooth muscle layer of pulmonary small vessels were measured using the Image J software.

EVG staining was performed using a EVG staining kit (ab150667, Abcam, Cambridge, UK) according to the manufacturer protocols to assess the elastic fibers of blood vessels. The elastic fiber contents were defined as the ratio of the positive area (dark blue area) to the total area. The vessel collagen fibers and the cardiac fibrosis were assessed using a Masson trichrome stain kit (ab150686, Abcam, Cambridge, UK) according to the manufacturer’s instructions. The collagen fiber content and the fibrosis extent were quantified using the ratio of the blue area to the total area. The area was measured using ImageJ software.

For immunofluorescence, after dewaxing, the slides were washed three times with phosphate-buffered saline, permeated with 0.5% Triton X-100 for 15 min, blocked with 10% donkey serum for 30 min, and stained with primary antibodies (anti-alpha smooth muscle actin (α-SMA) (ab7817, Abcam, Cambridge, UK) or anti-cardiac troponin T (cTnT) (ab459327, Abcam, Cambridge, UK)) overnight at 4 °C. After washing the slides 3 times, we incubated the sections or cells with secondary antibodies and 4’,6-diamidino-2-phenylindole (DAPI) for 30 min. The average intensities of α-SMA and cTnT were quantified using ImageJ software. To assess cardiomyocyte size, the slides were incubated with Alexa FluorTM 488 labeled-Wheat Germ Agglutinin (WGA) (W32466, Thermo Fisher Scientific Inc. Waltham, USA) for 10 min. The cross sectional area of the co-stained (cTnT and WGA) cells were measured for the statistical analysis.

### Statistical analysis

Continuous data were expressed as mean ± standard deviation. Differences were tested with Student’s *t*-test if the data were normally distributed; otherwise, they were tested with the rank sum test. *P* < 0.05 were considered to be statistically significant. Statistical analyses were performed using SAS software version 9.2 (SAS Institute Inc., Cary, NC, USA).

## Results

### Animal survival rate and body weight

Four pups (3 in the PVS group and 1 in the sham group) were unable to wake up after the surgery, indicating the lethality was higher in the PVS than that in the sham groups. There was no maternal cannibalization over the next 3 days, and 76 rats remained alive at P30. Therefore, the survival rate was 95%. At P21, the body weight (BW) in the PVS group was significantly reduced compared to that in the sham group (Additional file 1: Figure S1).

### PA acceleration time of PVS rats

**Fig. 2:**
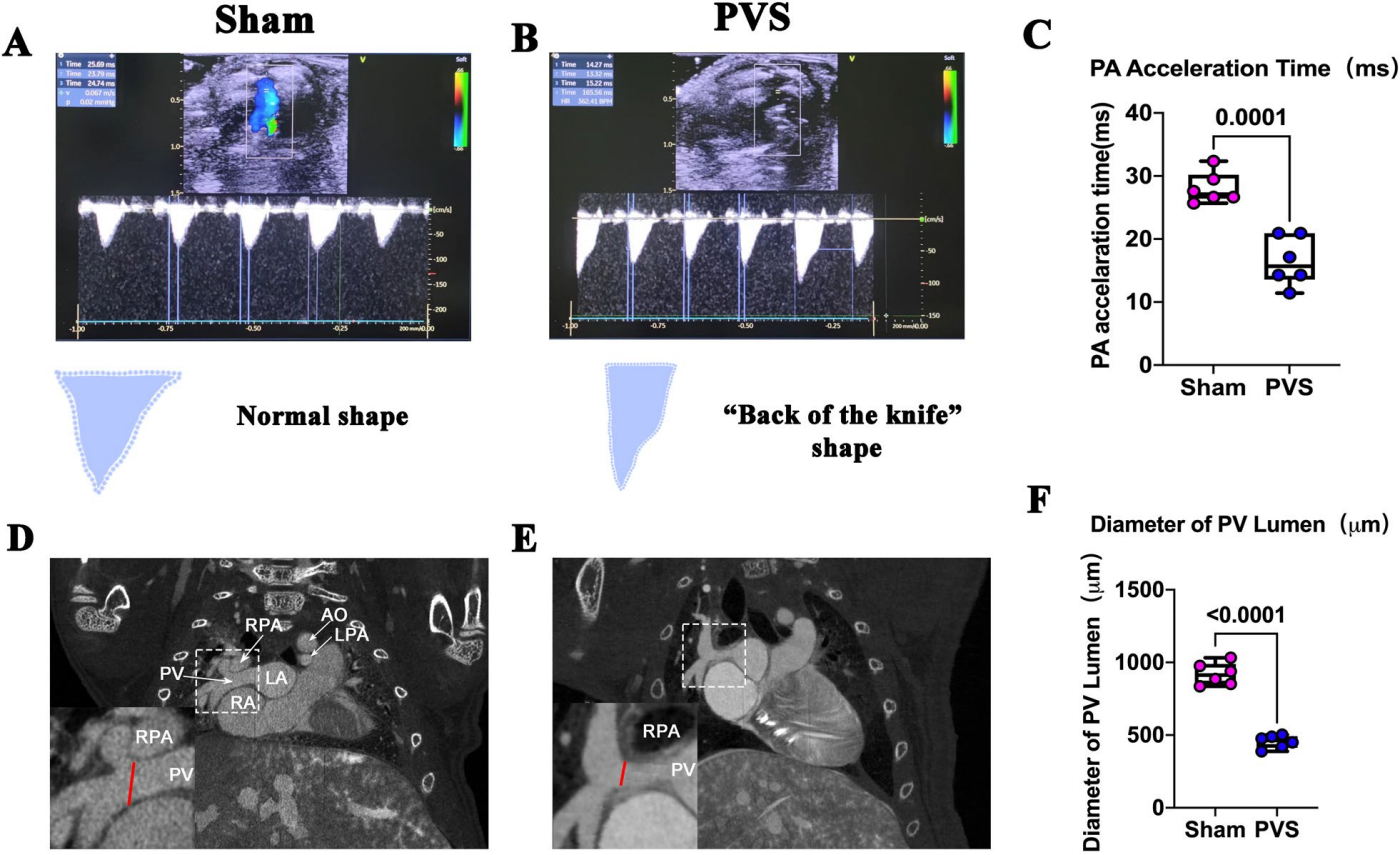
CT and echocardiography examinations of PVS rats. **A** Representative echocardiography of main PA from the sham group. The wave forms in pulsed-wave Doppler mode are triangle shaped. **B** Representative echocardiography of main PA from the PVS group. The wave forms in pulsed-wave Doppler mode are shaped like the back of a knife. **C** Quantification of PA acceleration time in the sham and PVS groups, *n* = 6. **D** Representative CT from the sham groups. *The length of the red line* indicates the PV lumen diameter at the banding site. *Ao* aorta; *RPA* right pulmonary artery; *LPA* left pulmonary artery; *LA* left atrium; *PV* pulmonary vein; *RA* right atrium. **E** Representative CT from the PVS groups. **F** PA lumen diameter quantification, *n* = 6

Because the PA acceleration time decreases in patients with PAH [17, 18], which is secondary to PVS [1, 7, 11, 12], we detected PA acceleration time to evaluate PAH and PVS at P21. As shown in Fig. 2A–C, the PVS group showed decreased acceleration time. Correspondingly, the wave form changed to a shape like the back of a knife (Fig. 2A and B) [17, 18]. These results suggest that there was PAH due to PVS, and that the PA acceleration time may serve as a simple and non-traumatic way to evaluate the model.

### Diameter of the PV lumen in PVS rats

To validate the presence of PVS, we performed CT at P21. As shown in Fig. 2D–F, the PV lumen diameter in the PVS group significantly decreased when compared to the sham group (*the red line* indicates the diameter of the PV lumen, 927.24 ± 82.69 μm vs 448.02 ± 20.08 μm in the sham and PVS groups, respectively, *p* < 0.01).

### Pulmonary congestion of PVS rats

**Fig. 3:**
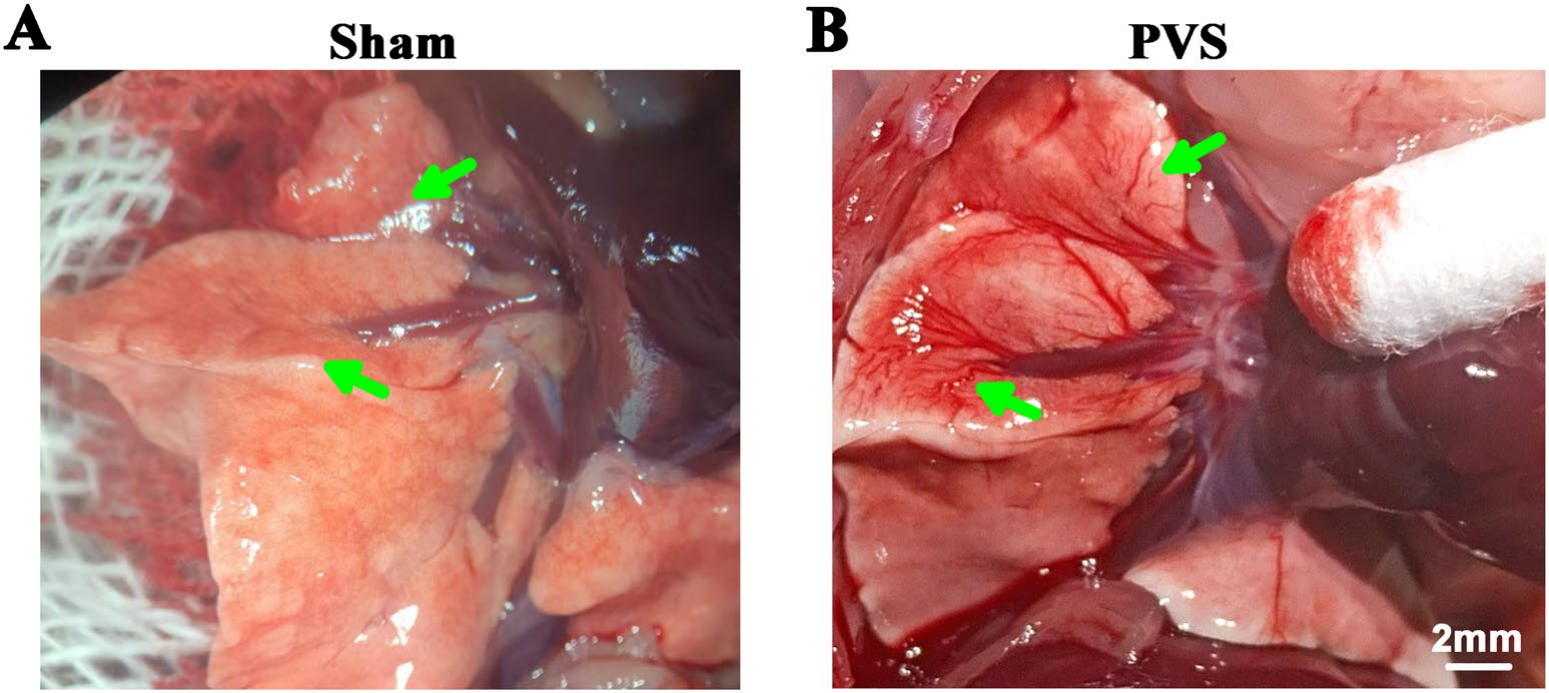
Gross lung images of PVS rats. **A** Representative gross lung image from the sham group. **B** Representative gross lung image from the PVS group. Note that there is pulmonary congestion in the anterior and middle lobes of the PVS group (*green arrows*)

To further confirm the PVS, we observed gross changes in the lungs. As shown in Fig. 3A and B, there was pulmonary congestion (*green arrows*) in the right anterior and middle lobes in the PVS group, while no pulmonary congestion was evident in the sham group (*green arrows*).

### Thickened upstream PV

**Fig. 4:**
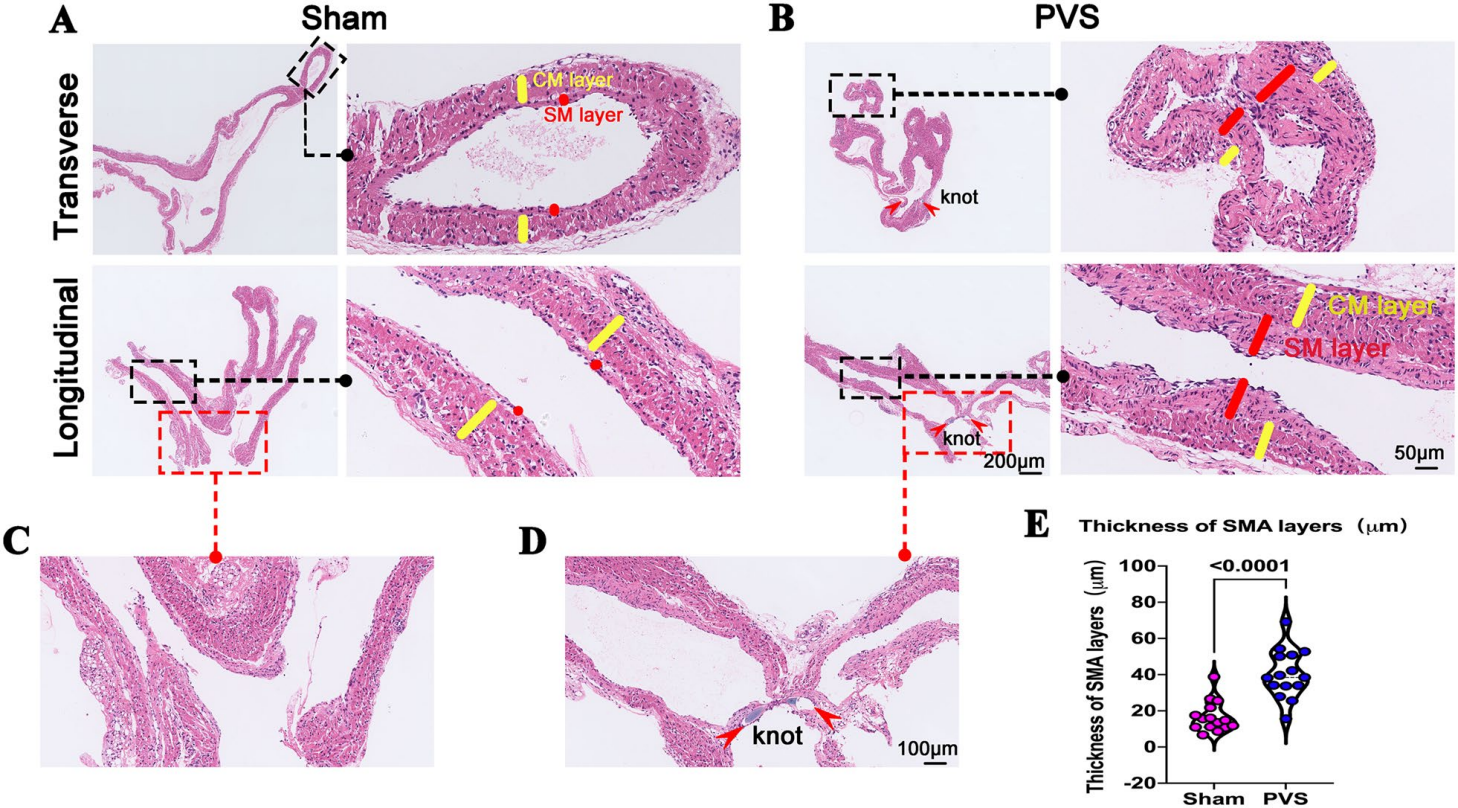
Thickened upstream PV of PVS rats. **A** Representative upstream PV and its local magnification in the sham group. *Red lines* indicate smooth muscle (SM) layer thickness; *yellow lines* indicate cardiomyocyte (CM) layer thickness; **B** Representative upstream PV and its local magnification in the PVS group. The red arrow indicates the banding knot; the red lines indicate the SM layer thickness; and the yellow lines indicate CM layer thickness. **C** Magnification of the PV from the sham group. **D** Magnification of the PV from the PVS group. The red arrow indicates the banding knot. **E** Quantification of the thickness of SM layer thickness, *n* = 30 slides from 6 rats

The major feature of PVS is intimal hyperplasia of the upstream PV, characterized by increased expression of smooth muscle cells [8, 10, 19]. Thus, we examined the intimal thickness of upstream PV in our PVS model. Figure 4A–E show that, as expected, the intimal thickness of upstream PV in the PVS group increased, with a significantly increased thickness of smooth muscle layer (Fig. 4A, B and E, *red line*). It should be noted that only the upstream PV expressed cardiomyocytes (Fig. 4A and B, *yellow line*), not the other veins [20].

To assess the elastic fibers and collagen fibers in the remodeling PV, we performed EVG and Masson staining. Additional file 2: Figure S2 shows that the elastic fibers and collagen fibers were primarily located at the middle wall of the PV. There was a significant increased content of elastic fibers and collagen fibers in the PVS group than in the sham group.

**Fig. 5:**
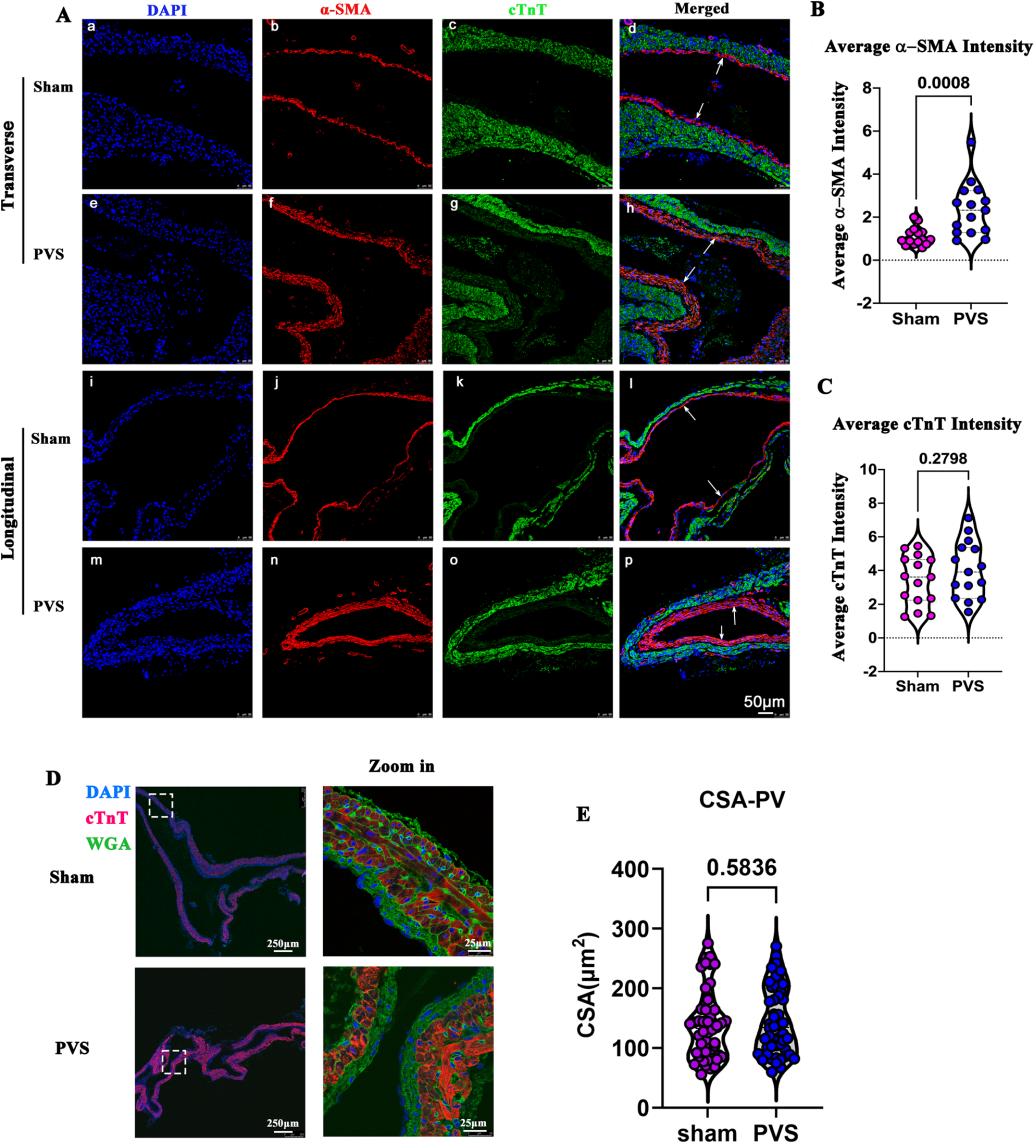
Immunofluorescence of the upstream PV. **A** Representative images from the sham and PVS groups. *White arrows* indicate the smooth muscle layer; α-SMA (*red*), cTnT (*green*), DAPI (*blue*). a–h represent the α-SMA and c-TnT staining of the upstream PV of sham and PVS rats in the transverse direction. i–p represent the α-SMA and c-TnT staining of the upstream PV of sham and PVS rats in the longitudinal direction. **B** Quantification of α-SMA intensity; *n* = 30 slides from 6 samples. **C** Quantification of cTnT intensity; *n* = 30 slides from 6 rats. **D** Representative WGA and c-TnT staining of PVs and the local magnification. WGA (green); c-TnT (red); DAPI (blue). The white dashed rectangle shows the original region of the magnification. **E** Quantification of the cross-sectional area (CSA) of cardiomyocytes, *n* = 25 slides from five rats

To further characterize the cell types of thickened upstream PV, we performed α-SMA and cTnT immunofluorescence staining to distinguish the smooth muscle cells and cardiomyocytes. As illustrated in Fig. 5A, the PVS group showed increased thickness of the smooth muscle layer and narrowed lumen (*white arrows)*. Correspondingly, as shown in Fig. 5B and C, the intensity of α-SMA significantly increased in the PVS group when compared to that in the sham group, while there were no significant differences in cTnT intensity between the two groups. WGA and cTnT staining showed that there were no differences in cardiomyocyte size between the two groups (Fig. 5D and E*)*. These results suggest that the intimal hyperplasia was the result of thickened smooth muscle layer but not of the cardiomyocyte layer.

### Lung remodeling of PVS rats

**Fig. 6:**
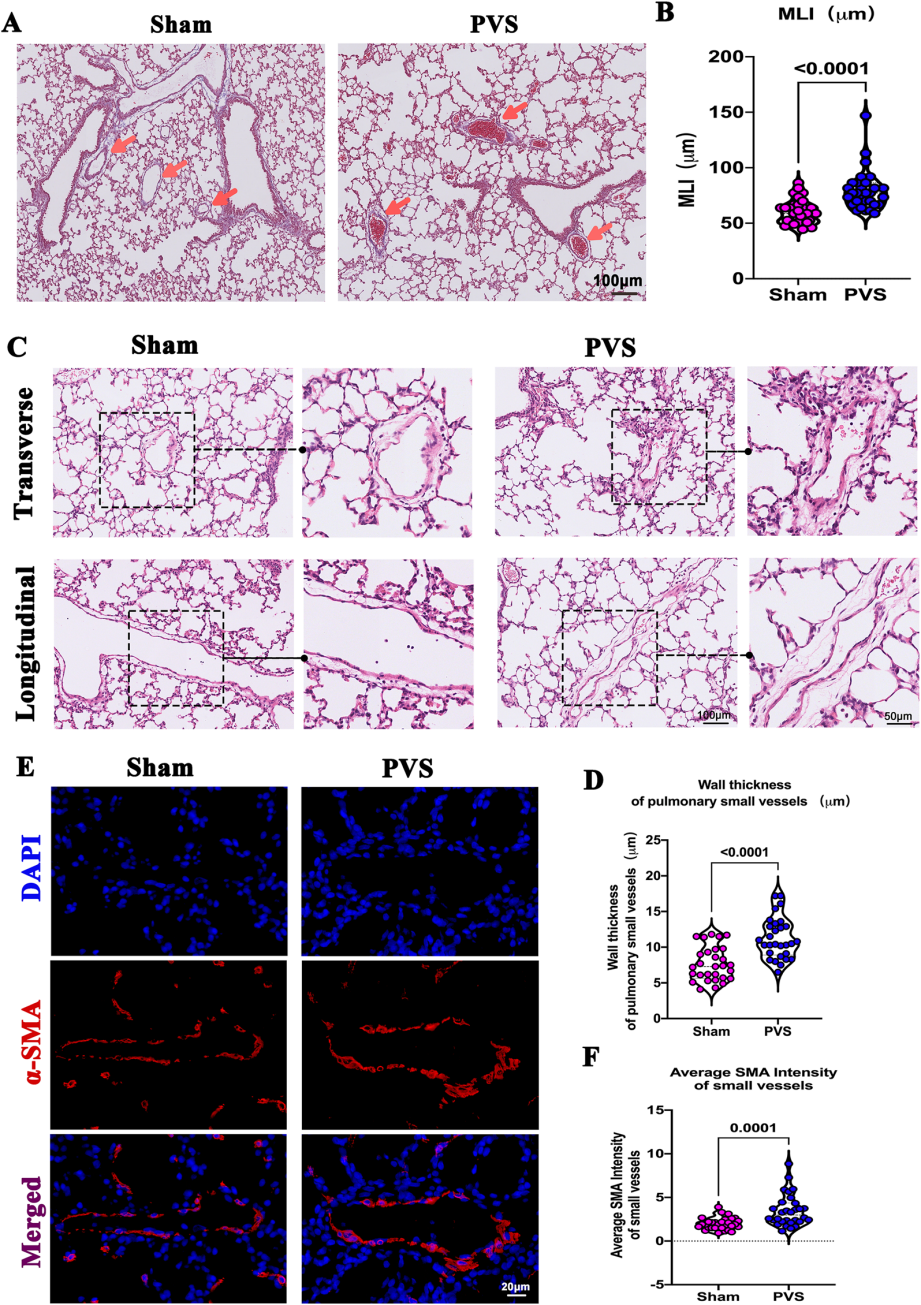
Lung remodeling in PVS rats. **A** Representative image of lung tissues from right anterior lobes; the red arrows indicate aggregation of red blood cells in the small vessels. **B** Quantification of MLI, *n* = 30 slides from 6 rats. **C** Representative images of the pulmonary small vessels. **D** Quantification of small vessel wall thickness, *n* = 30 slides from 6 rats. **E** Representative α-SMA staining of small vessels. α-SMA (red); DAPI (blue). **F** Quantification of α-SMA intensity, *n* = 30 slides from 6 rats

We looked for secondary effects to confirm the creation of PVS in our rat models. First, the histological changes in lung tissue were examined. As shown in Fig. 6A, there was aggregation of red blood cells in the small vessels of the PVS group, while none was observed in the sham group (*red arrows*). This was consistent with the gross lung sample examination (Fig. 3A and B, *green arrows*). In addition, the number of alveoli was reduced in the PVS group, as indicated by the increase in MLI (Fig. 6A and B), suggesting that PVS impaired postnatal alveolar development. As far as we know, this phenomenon has never been reported. The increased wall thickness of small pulmonary vessels, a defining characteristic of PAH, was also evidenced in the PVS group (Fig. 6C and D). Consistent with this finding, the α-SMA intensity of small pulmonary vessels in the PVS group increased (Fig. 6E and F). These results suggest that PVS caused extensive lung remodeling, which may account for the PAH.

### RV remodeling in PVS rats

**Fig. 7:**
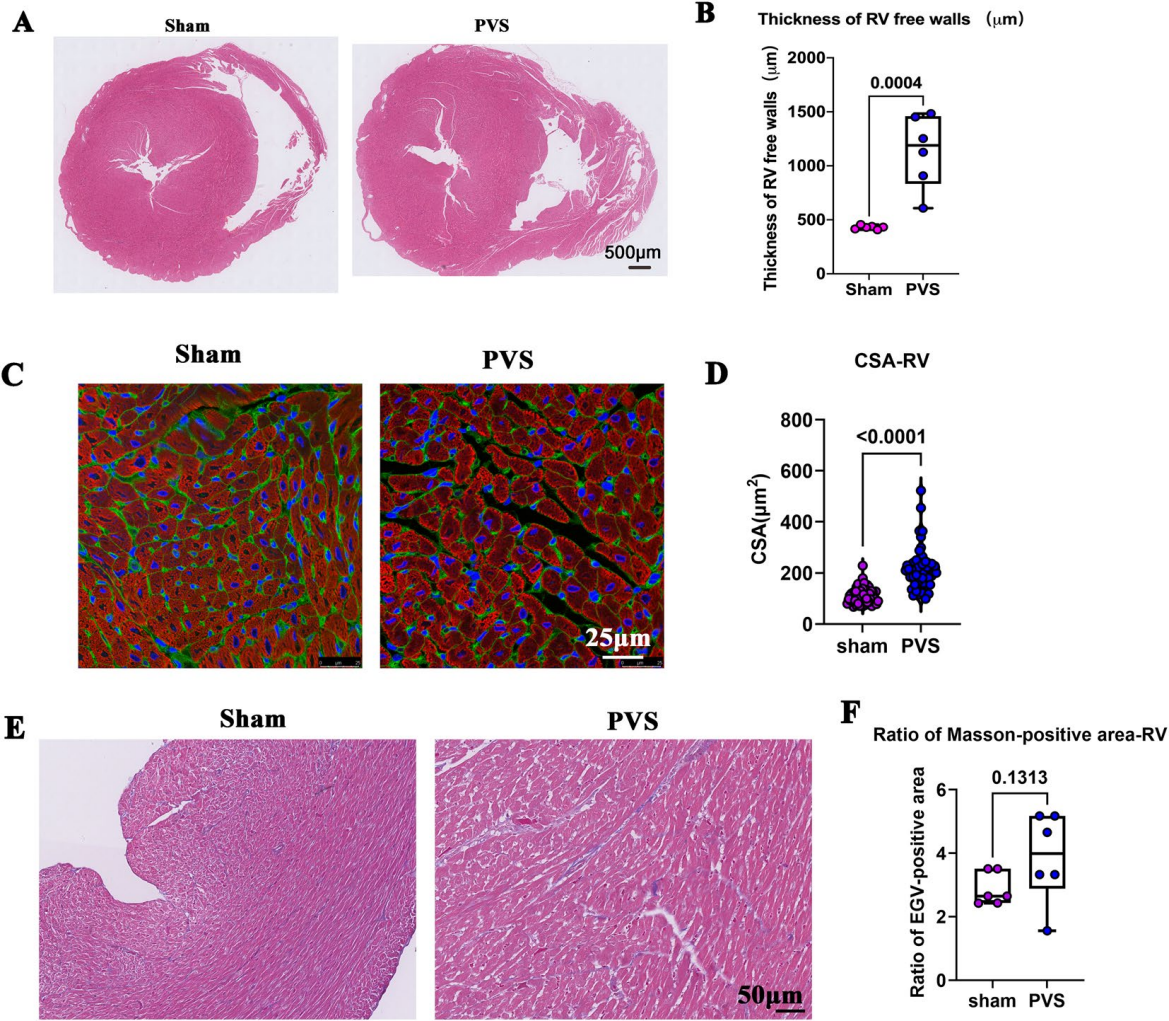
RV remodeling in PVS rats. **A** Representative H&E staining of RVs. **B** Quantification of RV free wall thickness, *n* = 6 rats. **C** Representative WGA and c-TnT staining of RVs. WGA (green); c-TnT (red); DAPI (blue). **D** Quantification of the cross-sectional area (CSA) of cardiomyocytes, n = 25 slides from five rats. **E** Representative Masson trichrome staining of RVs. **F** Quantification of the ratio of the fibrosis area, n = 6 rats

We examined the histological changes in RV tissue. As shown in Fig. 7A and B, the RV free wall thickness significantly increased in the PVS group compared to that of the sham group. Consistent with the increased RV wall thickness, the RV cardiomyocyte size also significantly increased in the PVS group (Fig. 7C and D). Further confirming RV remodeling under the PVS condition. However, we did not find increased fibrotic production in the remodeling RV (Fig. 7E and F), which is consistent with previous reports. These results indicated that the neonatal hearts had a strong compensatory ability and did not show fibrosis under the pressure overload condition [14].

## Discussion

Currently, PVS remains a highly challenging pediatric clinical issue [4]. Unlike adult PVS, which has an indolent developing course [21], pediatric PVS can be relentless and progressive, and can even cause neointimal obstruction of the pulmonary veins.

The etiologies and mechanisms of PVS are very elusive, inspiring us to develop the current rat model to enhance our understanding of these issues. Several possible contributory factors have been suggested in the literature, including genetic mutation, external compression from thoracic structures, local distortion of veins due to atelectasis, and local injury at the site of TAPVC repair [19].

Histological analyses of PVS lesions have found focal or diffuse lesions with myofibroblast-like cells (smooth muscle cells secreting extracellular matrix) and extracellular matrix deposition in human specimens [22, 23]. Studies on the surgical porcine model have suggested that transforming growth factor beta signaling, as well as the activation of other signaling pathways, may play roles in extracellular matrix deposition [20]. Myofibroblast deposition is a common feature of human PVS, porcine, and current rat models. However, therapies targeting myofibroblast deposition have yielded poor results [3]. Further investigation is required to determine where myofibroblasts come from and what their subcategories are, as well as whether there are any other cell types or mechanisms involved in PVS in addition to myofibroblasts. By using this newly created PVS model, our single-cell sequencing results suggested that there was a significant decrease in the proportion of endothelial cells and a significant increase in the proportion of smooth muscle cells and fibroblasts in the PVS group (data not shown). It is possible that the myofibroblasts may originate from the transformation of smooth muscle cells and fibroblasts, and the reduced endothelial cells may initiate inflammation. These events may also result in extracellular matrix deposition and myofibroblast activation. We hope that this introduction of a neonatal PVS rat model will advance our understanding of this condition and its treatment.

The current study created a successful neonatal rat model of PVS that will advance our understanding of PVS recurrence and improve its treatment. Compared to the piglet model of PVS, the rat model has several advantages: (1) the procedure for creating a neonatal rat PVS model does not exceed 10 min and a smaller space and a shorter breeding time are required for raising rats, resulting in lower time and money requirements; (2) a single skilled surgeon or trained student, instead of a team, is all that is needed for the procedure; and (3) genetic manipulation and drug evaluation are easier to perform with rats.

The neonatal rat PVS model may also aid in researching the secondary effects of PVS, the mechanisms of which are rarely reported [1–4]. Whether the PAH and RV failures caused by PVS are different from other types of PAH and RV failures should be explored, as well as whether the exocrine factors produced by the secondary effects of PVS induce the recurrence of PVS. A lineage tracing experiment on the neonatal rat PVS model could help identify the origin of myofibroblasts, which cause stenotic PV intimal hyperplasia.

Our study has a few limitations. First, we used horizontal thoracotomy, which damages the muscles that assist in breathing. However, in clinical practice, there is no relevant change in the muscles that assist breathing in children with PVS. A less invasive surgical approach that does not harm the breathing muscles should be developed in the future. Second, a long-term follow up of the model has not been made. Third, whether the banding size affects the progression of PVS has not been determined. Future neonatal mouse models of PVS will further advance our understanding of PVS.

Another limitation of this study was the difficulty of analyzing the hemodynamic parameters. To measure the RV function, magnetic resonance imaging (MRI) could be performed on seven-day-old rats, and the RV ejection fraction, along with other parameters, could then be acquired. Echocardiography could be performed on the four-chamber view of the heart in 21-day-old rats to visualize the blood flow in the PV. Because the banded PVs were located on the right side and converged to the left atrium, the blood flow across the connection of the PV and left atrium could be accelerated and recorded. Cardiac catheterization could also be performed on 30-day-old rats to assess the RV pressure.

In summary, the PVS rat model introduced in our study provides a sound platform for future studies of PVS and its effects.

## Supplementary Information

**Additional file 1: Figure S1.** Body weight of the rats in the sham and PVS groups at P21.

**Additional file 2: Figure S2.** Assessment of the elastic fibers and collagen fibers in the PV.Representative EVG staining of PVs and the local magnifications in the sham and PVS groups. The black dashed rectangle shows the original region of the magnifications.Representative Masson trichrome staining of the PVs and the local magnification in the sham and PVS groups. The black dashed rectangle shows the original region of the PV magnifications.Quantification of the ratio of the EVG-positive area, *n* = 6 rats.Quantification of the ratio of the Masson-positive area, *n* = 6 rats.

**Additional file 3: Video S1.** Surgical procedures of neonatal rat PVS model

## Data Availability

All of the data in the current study is available from the corresponding author upon reasonable request.

## Abbreviations

PV: Pulmonary vein
PVS: Pulmonary vein stenosis
PAH: Pulmonary arterial hypertension
RV: Right ventricular
H&E: Hematoxylin and eosin
PA: Pulmonary artery
CT: Computed tomography
H&E: Hematoxylin and eosin
α-SMA: Alpha smooth muscle actin
cTnT: Cardiac troponin T
MLI: Mean linear intercepts
