# Supplementary figures and images for: A neonatal rat model of pulmonary vein stenosis
Source: Cell Biosci. 2023 Jun 19;13:112. doi: 10.1186/s13578-023-01058-8 (PMC10278335; doi:10.1186/s13578-023-01058-8)

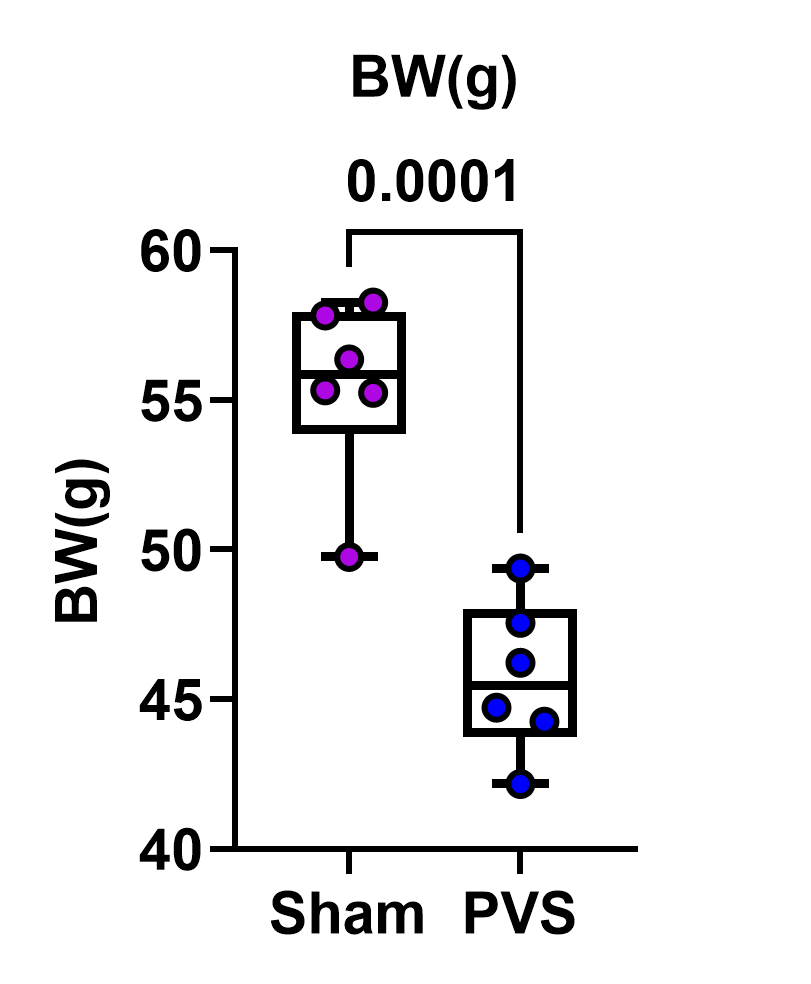

Supplement: Supplementary file 1 — Additional file 1: Figure S1. Body weight of the rats in the sham and PVS groups at P21. [file 13578_2023_1058_MOESM1_ESM.tif]
